# Late intraocular Lens dislocation following scleral depression: a case report

**DOI:** 10.1186/s12886-020-1327-3

**Published:** 2020-01-30

**Authors:** Maria V. Castanos, Tyler Najac, Jacqueline Dauhajre, Douglas F. Buxton

**Affiliations:** 1 New York Eye and Ear Infirmary of Mount Sinai, 310 E 14th St, New York, NY 10003 USA; 2 Lewis Katz School of Medicine at Temple University, 3500 N Broad St, Philadelphia, PA 19140 USA

**Keywords:** Intraocular lens, Dislocation, Late dislocation

## Abstract

**Background:**

The case describes a rare entity. Most cases of IOL dislocation are associated with surgical trauma or preexisting zonulopathy. This patient presents IOL dislocation following routine exam, suggesting the need of careful evaluation of zonular integrity on pseudopahkic patients.

**Methods:**

Patient is a 65 year old who presented with sudden loss of vision and pain following retinal examination using scleral depression. Patient was diagnosed with late intraocular lens dislocation, which was subsequently for proper repositioning of IOL.

**Conclusion:**

Pseduophakic eyes should be approached with caution when scleral indentation is attempted due to the possibility of zonular dehiscence and subsequent intraocular lens dislocation.

## Background

Intraocular lens dislocation is an uncommon complication of cataract surgery with an incidence between 0.2 to 2.8% [1]. Dislocations are divided into early and late cases.

Early intraocular lens (IOL) dislocations are due to improper IOL fixation and occur within the first 3 months following cataract surgery. Early dislocations are most commonly attributed to posterior capsule tears, often referred to as the sunset or sunrise syndrome [2]. Zonular rupture, known as in-the-bag dislocation, is also a main cause of early IOL dislocation. The zonule may be damaged intraoperatively due to posterior pressure on the lens or occur during IOL implantation, among other traumatic maneuvers [3].

Late, spontaneous IOL dislocations occur 3 months following cataract surgery. They are generally attributed to progressive zonular weakness after complicated or even uncomplicated cataract surgery. This type presents with an intact capsular bag [4]. It is characterized by an IOL that is adequately positioned within the capsular bag and the entire capsular-IOL complex decenters [5]. In his study, Kreptse et al. [6] Analyzed patients who were treated for IOL dislocation and found that 87.9% of late IOL dislocations exhibited an intact capsular bag and only 12% of late IOL dislocations were out-of-the-bag or with capsular bag defects. We present a case study of a 65-year-old male with a late, spontaneous IOL dislocation 8 years post-cataract surgery, following scleral depression.

### Case presentation

**Fig. 1:**
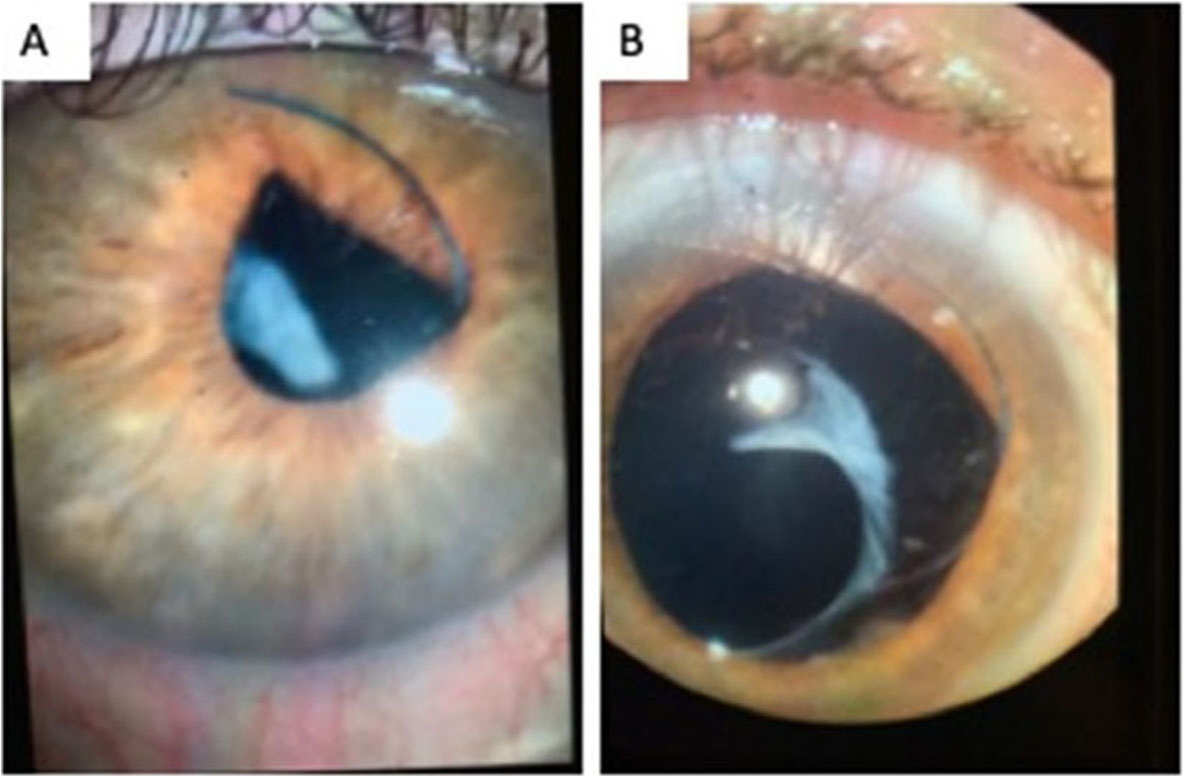
Photos taken of right eye showing dislocated intraocular lens. **a** Before dilation. **b** After dilation

An adult patient, with no relevant, medical history presented with sudden loss of vision and pain following retinal examination using scleral depression. He had undergone extraction in the right eye 8 years prior with a resulting visual acuity of 20/20. Onset of floaters in both eyes led to a retinal consultation. Immediately following scleral depression, which showed no tears or holes to ora serrata, the patient experienced immediate loss of vision and pain. After 3 days, the patient returned to his cataract surgeon, who diagnosed a posterior lens dislocation. Visual acuity was decreased to 20/80 associated with monocular diplopia. Slit lamp exam revealed 2+ cell and flare, and a dislocated superior haptic on the anterior face of the iris. (Fig. 1) Upon dilation, a 180° zonular dehiscence was noted and the whole posterior capsule-IOL complex decentered temporally. Vitreous prolapse was also recorded.

**Fig. 2:**
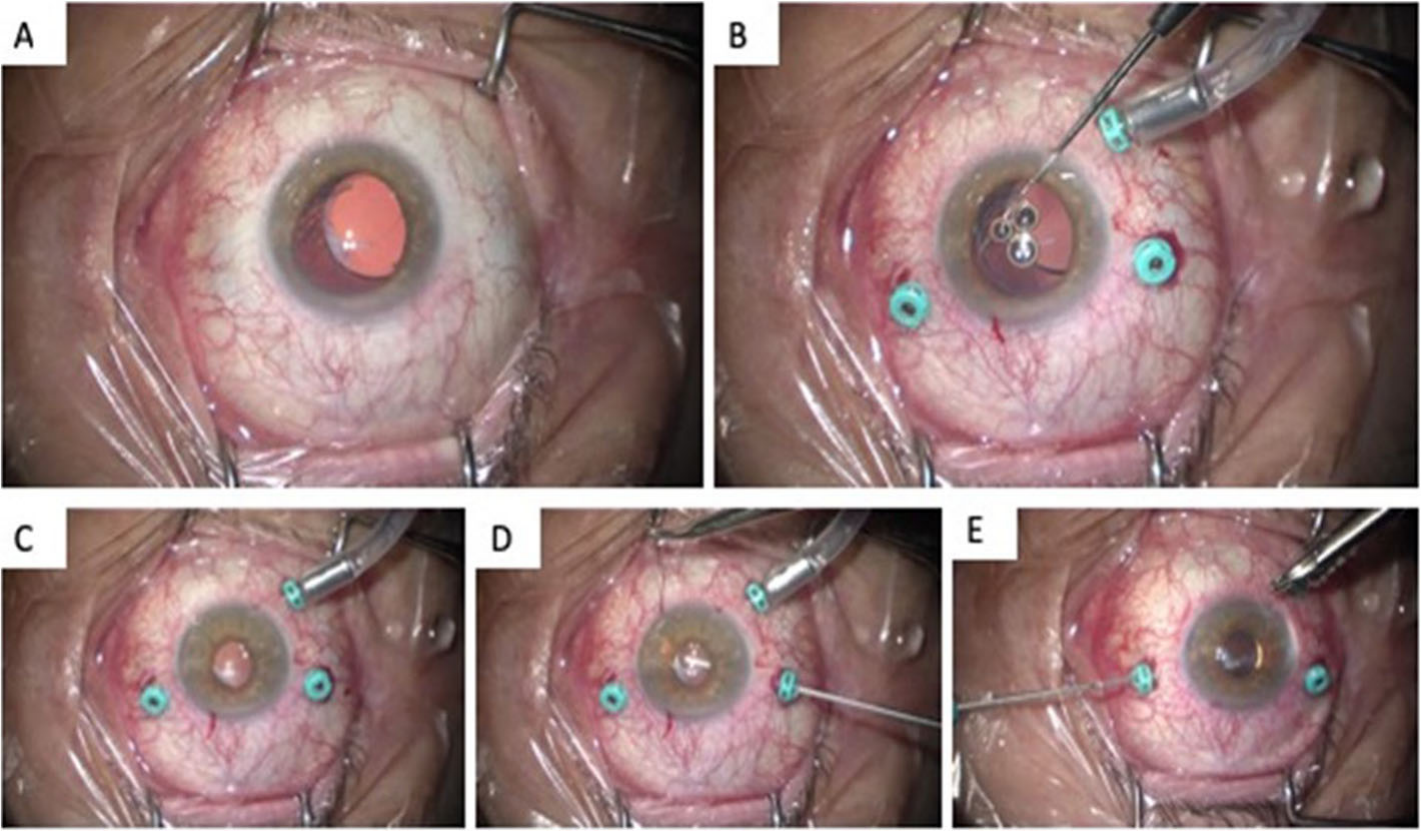
Intraoperative images. **a** Six o’clock zonular dehiscence of the right eye. **b** Repositioning of haptic using Kuglen hook. **c** View of lens after inducing miosis intraoperatively. **d** Suturing superior haptic to iris. **e** Suturing inferior haptic to the iris

A pars plana vitrectomy and IOL repositioning were carried out combined with fixation of both haptics to the iris with a McCannel technique. (Fig. 2) Postoperatively, the patient regained 20/20 vision and had no further complications.

## Discussion and conclusion

The first case of spontaneous in-the-bag IOL dislocation was recorded by Davidson in a patient with capsule contraction syndrome [7]. Late spontaneous dislocations represent a small subset of patients within the reported cases of posterior chamber IOL dislocations [8]. In recent years, late in-the-bag IOL subluxation has been reported more frequently.

Spontaneous IOL dislocations may occur several years following cataract surgery [9]. In their retrospective cohort study, Pueringer et al. [10] reviewed 14,471 cases of cataract extraction between 1980 and 2009 and discovered that 5, 10, 20, and 25 years after cataract extraction the risk of IOL dislocation was 0.1, 0.2, 0.7, and 1.7%, respectively [10]. The time from cataract surgery to repositioning surgery has ranged from 6.9 to 8.5 years. Fernandez et al. [9] found a mean time interval from cataract surgery to late dislocation of 7.5 ± 5.2 years when retrospectively reviewing 61 cases of late intraocular lens dislocation. Similarly, Davis et al. [11] reviewed 86 cases of late, in-the-bag, spontaneous intraocular lens dislocation and found postoperative time to dislocation to be similar among the entire population of pseudoexfoliation syndrome patients and vitreoretinal surgery patients, with mean time of 8.5 years. Krepste et al. [6] found intervals were shorter for eyes with zonular laxity, complicated cataract surgery, uveitis, mature cataracts, older age, and zonular dehiscence. Some authors have reported cases as late as 18 years after surgery [12].

Fernandez Buenaga et al. [9] found in his review of 61 cases that patients’ mean age was 63.5 years at time of cataract surgery and 71.2 years at time of explantation surgery. The mean age decreased in patients with high myopia at time of surgery and at time of explantation surgery, 52.9 and 65 years, respectively. The author found a higher incidence among males (68.9%) than in females, as did Hayashi et al. [13] and Gross et al. [1]

Different etiologies have been postulated in postoperative capsule dislocation: zonular dehiscence, capsular contraction syndrome, and surgical and postoperative trauma [5].

- Zonular dehiscence progresses slowly following cataract surgery, normally due to unrecognized or subclinical zonular damage prior to surgery. Zonulysis likely develops slowly over time because surgeons rarely report intraoperative phacodonesis [11]. Breyer et al. [14] described 5 patients and Jehan et al. [15] described 8 patients who presented with late, spontaneous dislocation associated with pseudoexfoliation. Pseudoexfoliation is known to be associated with progressive zonulopathy, a degenerative process at the interface of the zonule to the basement membrane of the ciliary processes. Compromised zonular ligaments can become vulnerable to external maneuvers and rupture. In the study presented by Wilson et al., postmortem histopathology on 27 eyes following extracapsular cataract extraction, 5 eyes demonstrated zonular disruption [3].
- Contraction of the capsular bag or “capsular contraction syndrome” leads to additional stress on the zonule that may or may not be already weakened postoperatively. The routine adoption of continuous curvilinear capsulorrhexis (CCC) has increased the risk of capsular contraction. According to Gimbel et al. [4], in-the-bag IOL dislocation were virtually non-existent prior to the advent of CCC. After surgery, some degree of capsular contraction may take place, reducing the aperture of the capsulotomy and shrinking the capsular bag’s diameter [1]. CCC induces capsular fibrosis, resulting in capsular contraction despite zonular support. The syndrome has mostly been described in patients with pseudoexfoliation, diabetes mellitus, and uveitis [5].
- Trauma or mechanical stress has also been studied as a cause of late lens dislocation in the setting of an intact capsular bag. Yamazaki et al. [16] reported an intraocular lens subluxation in a patient with facial atopic dermatitis. These authors postulated that zonular rupture occurred due to pressure exerted by persistent eye rubbing. The trauma induced rupture of all the zonular fibers and subsequent luxation of the capsular bag IOL complex. In a case reported by Zech et al. [17], a patient presented with IOL subluxation with an intact capsular bag due to ocular contusion. Although a rare presentation, trauma in the setting of a weakened zonule may lead to late dislocation. Additionally, Gross et al. [1], in a retrospective analysis of 25 eyes with lens dislocation, found that 16% were associated with a traumatic event. Similarly, Kreptse et al. [6] showed that 21.6% of in-the-bag lens dislocations were attributed to trauma.

Many factors have been linked to late intraocular lens subluxation with an intact capsular bag, including aging, high myopia, pseudoexfoliation, trauma, previous vitreoretinal surgery, diabetes mellitus, connective tissue disease, acute angle glaucoma, and retinitis pigmentosa. These factors have a common effect; they increase zonular weakness and capsular contraction [5]. Pseudoexfoliation appears to be the most common risk factor. Ostern et al. [18] showed that after cataract extraction surgery, most intraocular lenses were found to be positioned more inferiorly in pseudoexfoliation patients than in controls, suggesting pre-existing zonular weakness.

The type of posterior chamber IOL may also be associated with the subsequent development of late IOL dislocation. In Lorente et al. [19], a retrospective analysis of 45 cases of intraocular lens dislocation, 25 eyes received a 3-piece acrylic IOL. Other studies found 1-piece PMMA IOLs to be the most commonly dislocated. Furthermore, Davis et al. [11] described PMMA IOLs in 28 cases, silicone IOLs in 33 cases, and hydrophobic acrylic IOLs in 25 cases, proving that all types of IOLs may be involved. In regards to material, it has been shown that plate haptic, silicone IOLs are more frequently associated with capsular contraction secondary to anterior capsule opacification than with acrylic, hydrophobic IOLs [19, 20].

Management of late IOL dislocation with an intact capsular bag involves either IOL repositioning or replacement. Overall, a surgical approach is recommended when any dislocation is detected. Unlike in cases of out-of-the bag IOL dislocations with relatively intact zonular integrity and adequate capsular support, in-the-bag dislocations invariably require suturing to iris or sclera due to concomitant, severe zonulopathy [19]. The approach depends on surgeon’s preference and the specific clinical features of individual cases, including type of IOL, presence of capsule tension ring (CTR), site of IOL dislocation, and other ocular co-morbidities.

The advantage of repositioning and suturing the dislocated IOL, rather than exchanging the lens, is reduced ocular and specifically endothelial trauma, and less postsurgical astigmatism due to a smaller incision [4]. Replacement is considered in advanced dislocations, damaged IOLs or haptics, and eyes with plate haptics IOLs with no CTR. In a recent review of the American Academy of Ophthalmology, there was no evidence to support the superiority of scleral-supported PCIOLs over open loop anterior chamber IOLs (AC IOLs) [21]. Kwong et al. [22] recently reported that results in eyes with AC IOLs were actually superior to scleral sutured IOLs in terms of postoperative BCVA. Lorente et al. [19] and Sarrafizadeh et al. [23] found that postoperative visual acuity between eyes with repositioning and replacement had no statistically significant difference.

To our knowledge no previous case has being reported of in-the-bag IOL dislocation precipitated by scleral indentation. Scleral indentation or scleral depression is a technique used to examine the peripheral fundus by inwardly displacing tissue and allowing stereoscopic examination of the peripheral retina. It is indicated in patients with symptoms of retinal detachment, history of blunt trauma, high axial myopia, aphakia, and retinal abnormalities such as holes and breaks. The technique is contraindicated in recent intraocular surgery, recent hyphema, or in suspected penetrating injuries or ruptured globes. It should be done with caution in patients with advanced glaucoma and in patients with intraocular lenses [24]. In a study where 20 healthy volunteers underwent scleral depression, statistically significant elevation of intraocular pressure from baseline was recorded. The subjects demonstrated a mean increase of 24.2 mmHg–27.5 mmHg at two and four minutes, respectively [25]. Few reports exist of complications following scleral indentation. Mercieca et al. [26] presented a patient with undiagnosed pellucid marginal degeneration who suffered a corneal perforation following scleral indentation.

It is our recommendation that careful inspection for zonular dehiscence be performed at slit lamp biomicroscopy before scleral indentation is attempted, especially in pseudophakic eyes. Three-mirror gonioscopy is a viable alternative should a zonular dehiscence be suspected. Our case study, although exceedingly rare, supports our recommendations.

## Data Availability

N/A

## Abbreviations

AC IOLS: Anterior chamber intraocular lens
CCC: Continuous curvilinear capsulorrhexis
CTR: Capsule tension ring
IOL: Intraocular lens
